# Supplementary material for: Pharmacological inhibition of CLK2 activates YAP by promoting alternative splicing of AMOTL2
Source: bioRxiv. 2023 Apr 19:2023.04.19.537449. Preprint. [Version 1] doi: 10.1101/2023.04.19.537449 (PMC10153145; doi:10.1101/2023.04.19.537449)
Supplement: Supplementary file 1 [file NIHPP2023.04.19.537449v1-supplement-1.pdf]

bioRxiv preprint doi: https://doi.org/10.1101/2023.04.19.537449; this version posted April 19, 2023. The copyright holder for this preprint (which was not certified by peer review) is the author/funder, who has granted bioRxiv a license to display the preprint in perpetuity. It is made available under aCC-BY 4.0 International license.

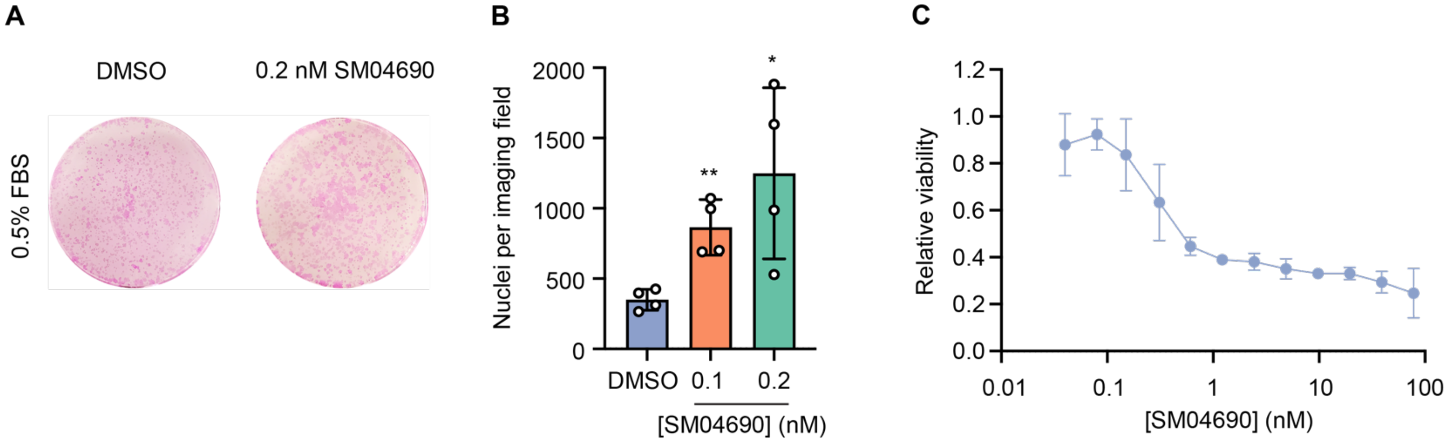

Figure S1. SM04690 induces YAP dependent proliferation at non-toxic concentrations. (A) Representative Rhodamine B staining, and (B) number of cells of human keratinocytes (HaCaT) in 0.5% FBS treated with SM04690 for 7 days (n=4, mean and s.d.). Statistical test is a univariate two-sided t-test calculated using GraphPad Prism (* P <0.0332, ** P < 0.0021). (C) Cell viability dose-response of SM04690 in 293A-TEAD-LUC cells treated for 72h with SM04690 evaluated using CellTiter-Glo luminescent cell viability assay (n=3 biological replicates, mean and s.d.).

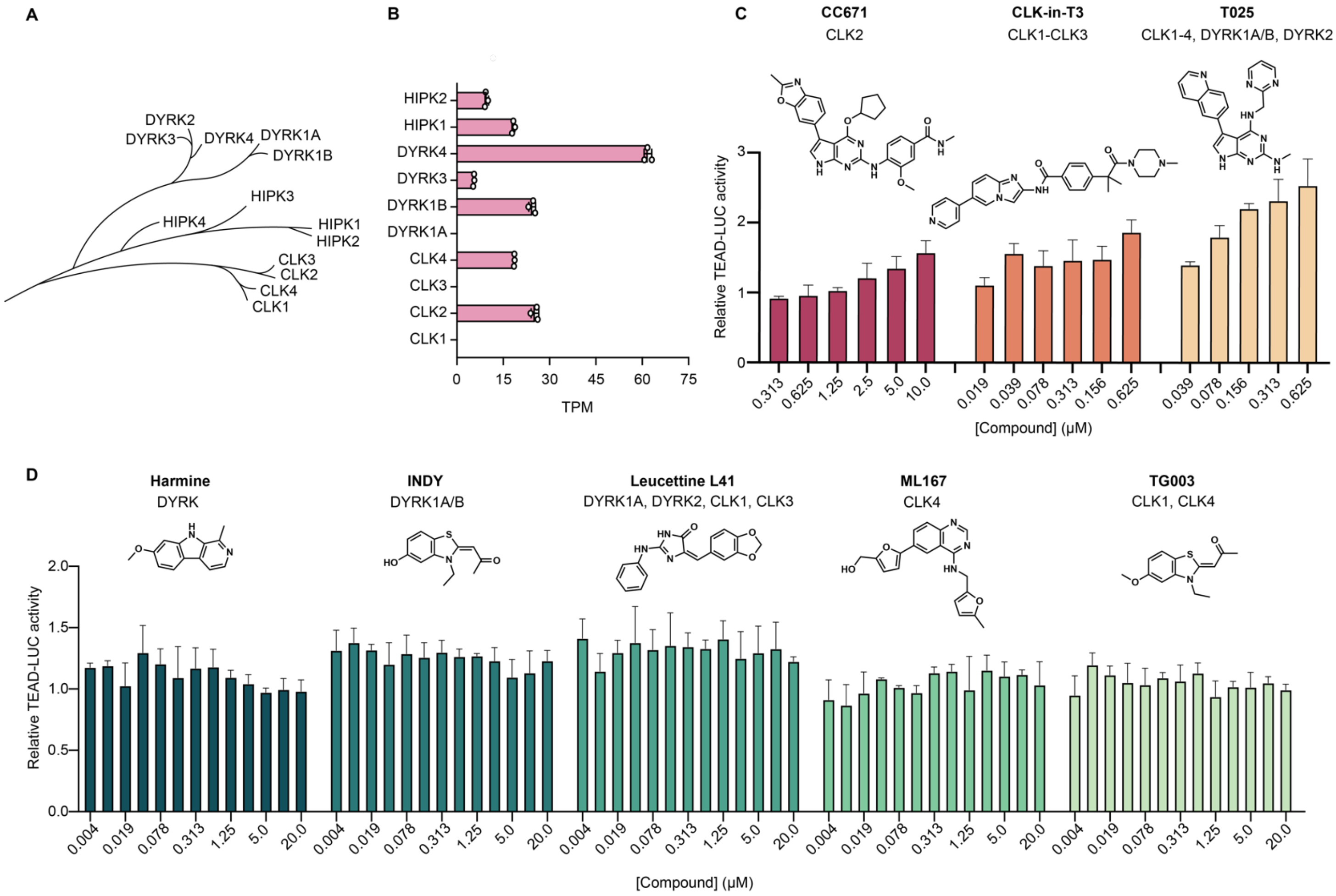

Figure S2. CLK2 inhibition activates YAP. (A) Family tree of CLK, DYRK, and HIPK kinases. (B) Transcripts per million (TPM) of the transcripts corresponding to reported targets of SM04690 in HEK293A cells, as determined by RNA-sequencing of unstimulated cells. (C) Relative TEAD-LUC activity of CLK2-targeting inhibitors CC671, CLK-in-T3, and T025 in cells treated for 24h with compound (n=3 biological replicates, mean and s.d.). (D) Relative TEAD-LUC activity of non-CLK2-targeting inhibitors Harmine, INDY, Leucettine L41, ML167, and TG003 in cells treated for 24h with compound (n=3 biological replicates, mean and s.d.).

bioRxiv preprint doi: https://doi.org/10.1101/2023.04.19.537449; this version posted April 19, 2023. The copyright holder for this preprint (which was not certified by peer review) is the author/funder, who has granted bioRxiv a license to display the preprint in perpetuity. It is made available under aCC-BY 4.0 International license.

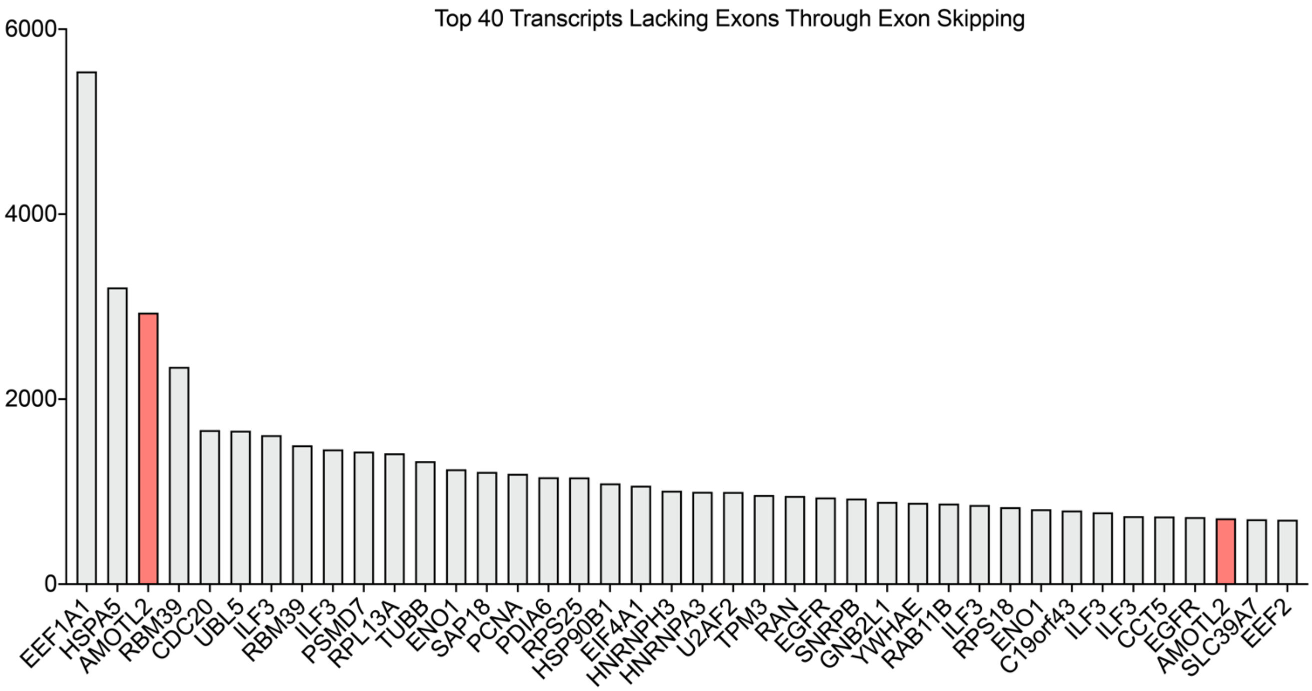

Figure S3. Top 40 transcripts with skipped exons in response to CLK2 inhibition. Number of skipped exons of the indicated transcripts in response to CLK2 inhibition. Figure derived from Araki, S. et al., *PLOS One*, 2015.

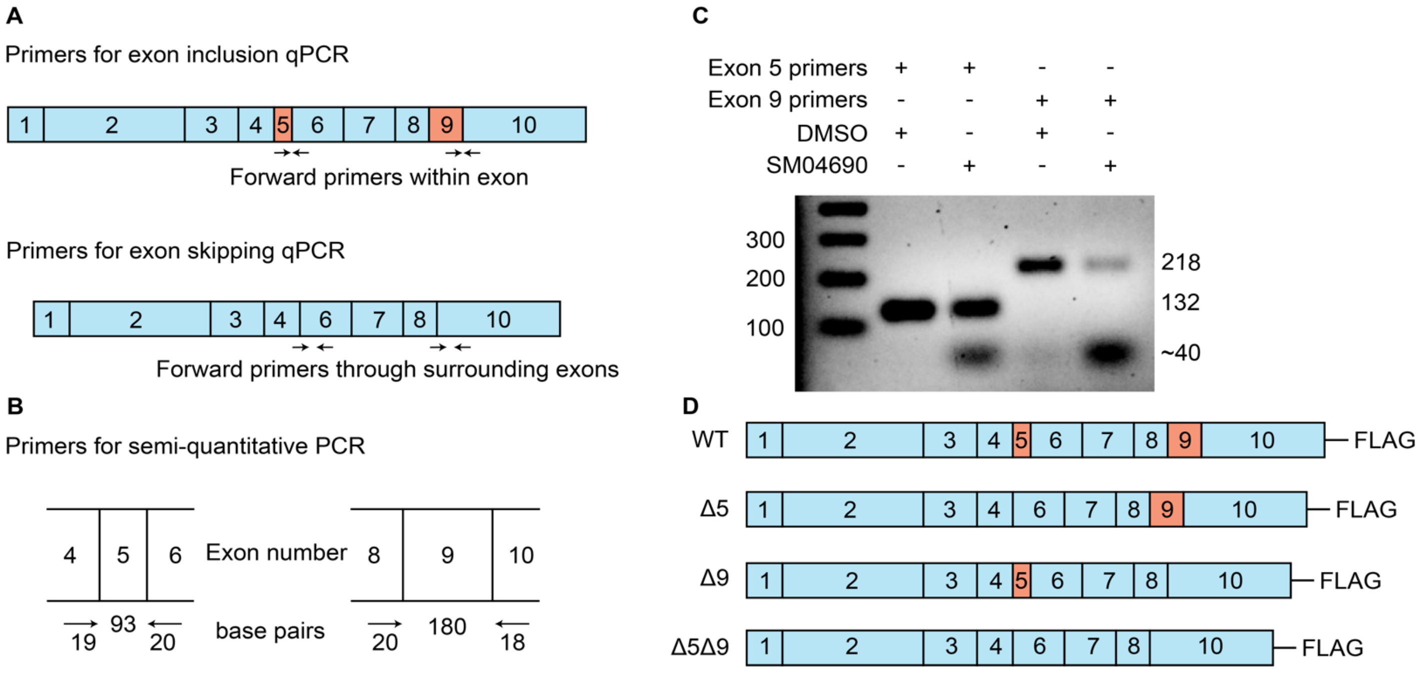

Figure S4. Inhibition of CLK2 by SM04690 causes alternative splicing of AMOTL2 exons 5 and 9. (A) Schematic of primer design to determine the presence of exon skipping in AMOTL2 by RT-qPCR. Exon inclusion primers were designed with the forward primer within the exon and the reverse primer at the start of the next flanking exon. Exon skipping primers were designed with the forward primer spanning the junction between the two exons flanking the skipped exon and the reverse primer within the adjacent exon. (B) Schematic of primer design to determine exon skipping in AMOTL2 by semi-quantitative PCR. The forward primer resides in the exon preceding the skipped exon and the reverse primer resides in the exon following the skipped exon. (C) Agarose gel electrophoresis of PCR amplified products using exon-specific primer sets. The primers amplified differently sized PCR amplicons depending on whether the template included the variable exon. Exon-included samples

bioRxiv preprint doi: https://doi.org/10.1101/2023.04.19.537449; this version posted April 19, 2023. The copyright holder for this preprint (which was not certified by peer review) is the author/funder, who has granted bioRxiv a license to display the preprint in perpetuity. It is made available under aCC-BY 4.0 International license.

appear in the gel at the size of the exon plus the size of the primers. Exon-skipped samples appear in the gel at the size of only the primers (~40 base pairs). (D) Schematic of C-terminal FLAG-tagged AMOTL2 constructs.

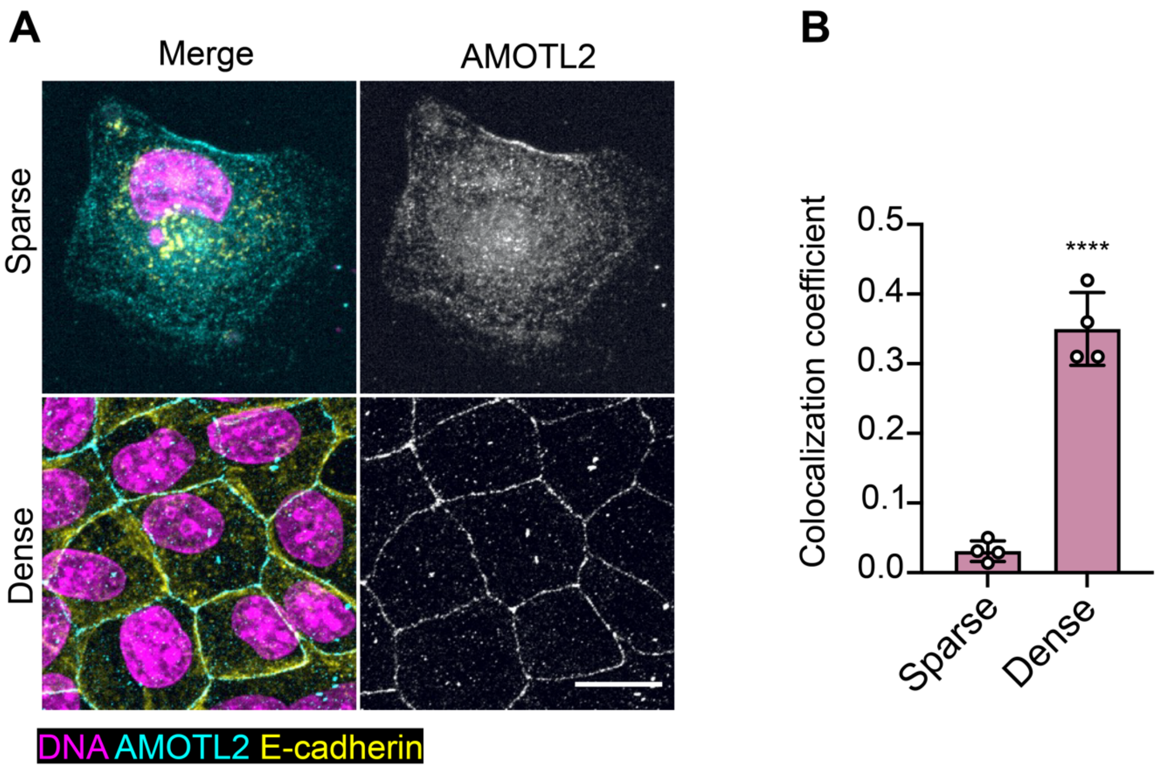

Figure S5. AMOTL2 displays density dependent localization to the plasma membrane. (A) Representative images of anti-AMOTL2 (teal) and anti-E-cadherin (yellow) immunofluorescent staining of MDCK cells grown in sparse (10,000 cells) and dense (400,000 cells) cell conditions with Hoechst 33342 (pink) to visualize nuclei (scale bar = 20 µm). (B) Quantification of anti-AMOTL2 and anti-E-cadherin correlative immunofluorescent staining (n=4, mean and s.d.). Statistical analysis is a univariate two-sided t-test calculated using GraphPad Prism (**** $P < 0.0001$).

bioRxiv preprint doi: https://doi.org/10.1101/2023.04.19.537449; this version posted April 19, 2023. The copyright holder for this preprint (which was not certified by peer review) is the author/funder, who has granted bioRxiv a license to display the preprint in perpetuity. It is made available under aCC-BY 4.0 International license.

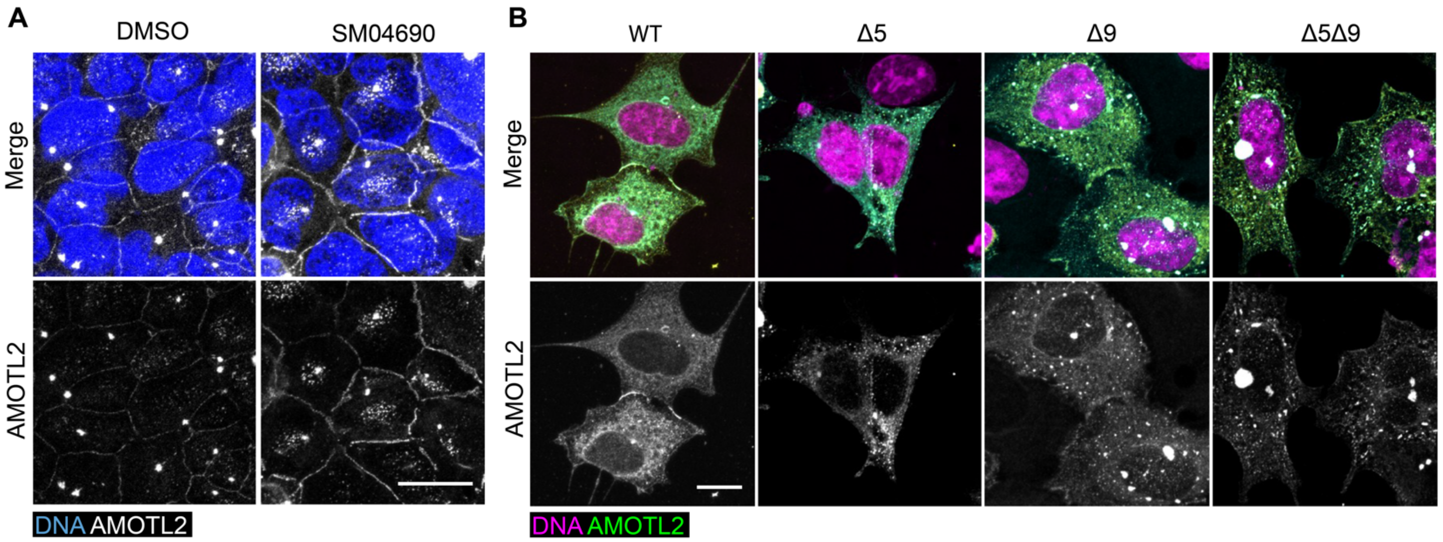

Figure S6. AMOTL2 spliceoforms are aggregation prone and do not localize to membranes. (A) Representative images of anti-AMOTL2 (white) immunofluorescent staining of HEK293A cells treated with control or 1μM SM04690 for 24h with Hoechst 33342 (blue) to visualize nuclei (scale bar = 20 μm). (B) Representative images of anti-AMOTL2 (green) immunofluorescent staining of HEK293A cells overexpressing AMOTL2 spliceoforms with Hoechst 33342 (pink) to visualize nuclei (scale bar = 10 μm).

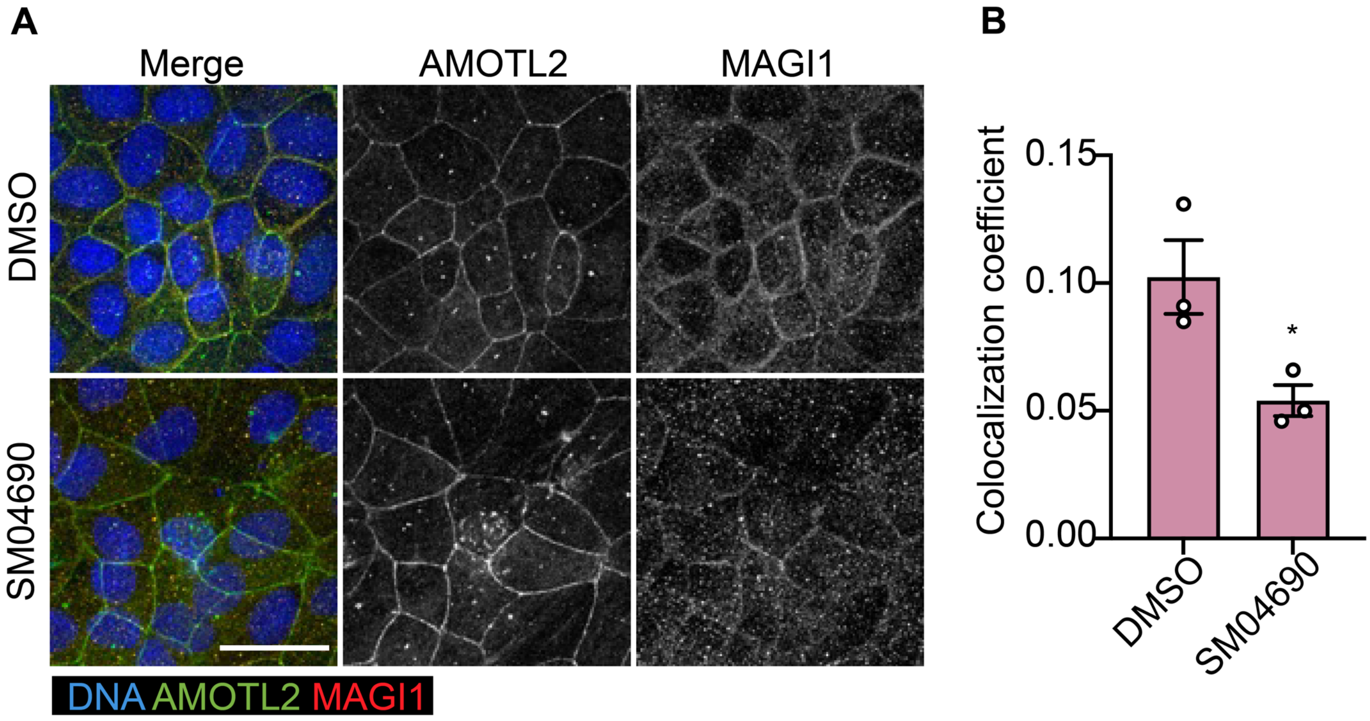

Figure S7. SM04690 treatment delocalizes AMOTL2 from MAGI1. (A) Representative images of anti-AMOTL2 (green) and anti-MAGI1 (red) immunofluorescent staining of MDCK cells treated with control or 1 μM SM04690 for 24h. Hoechst 33342 (blue) was used to visualize nuclei (scale bar = 30 μm). (B) Quantification of anti-AMOTL2 and anti-MAGI1 correlative immunofluorescent staining (n=3, mean and s.d.). Statistical analysis is a univariate two-sided t-test calculated using GraphPad Prism (**P* <0.0332).
